# Intraoperative Complications During Orthopaedic Spinal Surgery in a Polypharmacy Patient With Multiple Comorbidities

**DOI:** 10.7759/cureus.39949

**Published:** 2023-06-04

**Authors:** Laurence Stolzenberg, Mohammad Usman, Austin Huang, Mohammad Ibrahim, Colby Kihara, Brandy M Bodiford

**Affiliations:** 1 Orthopaedic Surgery, Alabama College of Osteopathic Medicine, Dothan, USA; 2 Anesthesiology, Alabama College of Osteopathic Medicine, Dothan, USA; 3 Neurology, Alabama College of Osteopathic Medicine, Dothan, USA; 4 Health Sciences, University of Central Florida, Orlando, USA; 5 Research, Alabama College of Osteopathic Medicine, Dothan, USA; 6 Family Medicine, Alabama College of Osteopathic Medicine, Dothan, USA

**Keywords:** alabama, spinal surgery, cervical arthroplasty, comorbid obesity, bone smash, orthopedic anesthesia, general internal medicine, pharma, ortho surgery, orthopaedic surger and rehabilitation

## Abstract

Orthopedic spinal surgeries, such as laminectomies or decompressions, have the potential to significantly increase quality of life for patients suffering from a spectrum of health issues ranging from neuropathy to chronic pain. Patients suffering from neurological symptoms such as weakness or neuropathy may lose significant function and become unable to perform the activities of daily living, however these delicate surgical interventions also come with significant risks to the health and well-being of those same patients. This is especially true with patients who have predisposing health conditions. Here, we discuss the effects of surgery on a patient with severe obesity, multiple confounding pre-existing conditions, and significant polypharmacy. An initially unremarkable spinal laminectomy and decompression surgery resulted in severe intraoperative complications that necessitated direct admission to the intensive care unit for significant post-operative management before he was able to be discharged safely. Although not a case of incredible rarity, we hope it can contribute to the growing body of data about the role of predisposing health conditions and polypharmacy in calculating and understanding the risks of orthopaedic surgery.

## Introduction

Orthopedic spinal surgeries are complex and delicate operations, with both serious benefits and risks. They are also extremely common in the United States with thousands of procedures performed every year. Even more notable is that the number of spinal surgeries increased by over 276% between 2002 and 2014 [1]. These surgical interventions are predicted to rise in quantity performed, as are all other orthopedic interventions with one million surgeries per year noted currently. Furthermore, the average orthopedic surgeon already performs over 30 procedures every month, and as such the increased volume will likely further strain the system [2]. As the population in the U.S. ages and obesity becomes more prevalent, the need for orthopedic procedures will continue to grow at a rapid rate. Therefore, these predisposing factors will continue to make postoperative care more complex in addition to there being more complicated cases in general [3-6]. 

There are several common and chronic health issues that are linked to significantly increased risks of complications to any procedure. There is convincing evidence that obesity is linked to worse surgical outcomes [3,4]. Notably, a large meta-analysis demonstrates that on a pool of 7,751 individuals with obesity (approximately 17% of which were considered morbidly obese), there was a strong association with an increased risk of mortality, longer stay in the intensive care unit (ICU), and overall higher rates of complications [3]. Hypertension is another significant risk factor, as it is associated with an increased risk of cardiovascular events, cerebrovascular events, bleeding and overall mortality [5]. Existing literature emphasizes the need to control hypertension prior to any major elective noncardiac or cardiac surgery, to minimize risk for adverse outcomes [5]. Diabetes mellitus is another well-studied perioperative risk factor that is commonly found in the general population. Studies have been completed specifically on outcomes in orthopedic surgeries and diabetes mellitus [6]. When combining the population level increases in these predisposing health conditions, it is reasonable to assume that we can expect the average surgical patient to become more complex as time passes. 

Polypharmacy is another major factor that can drastically increase the odds of a poor postoperative outcome. Polypharmacy is generally defined as taking five or more medications on a continuous basis to treat chronic diseases of all kinds, or two or more medications with significant interactions [7]. While polypharmacy is often routine in elderly patients with many chronic issues, it is also a reality for many younger individuals as well. As every medication has its own long list of potential adverse effects and interactions, the reality is that taking many medications often can result in unforeseen complications or adverse outcomes in surgery. Polypharmacy has been shown in previous literature to be linked to poor post-operative outcomes in elderly patients, but it has not been adequately researched in other populations despite the increased prevalence [8]. 

These increasingly common predisposing health conditions lead to an expected increase in the risk of complications that can be experienced during and after orthopedic surgery. This may even be the case for procedures that initially may have been considered low risk by the surgeon when they make the decision to operate [3,4]. As the number of surgical procedures continues to grow, it is vitally important that proper care is taken to clear complicated patients for the operating table. It is also critical to ensure that proper informed consent is obtained with the patient's full understanding of both the significant benefits of orthopedic surgery but also the fundamental risks. In this report, we present a case of a patient with many of these predisposing health conditions who suffered intraoperative complications during spinal surgery. Hopefully, this case can be included in further studies used to provide further guidance on the possible consequences and complications of orthopedic surgery in a patient suffering from these conditions. 

## Case presentation

This patient was a 55-year-old male who presented to the operating room (OR) for an elective laminectomy and decompression by an orthopaedic surgeon. The procedure was initially indicated because of severe lumbar radiculopathy. The patient was morbidly obese with a weight of 149 kilograms (kg) and a Body Mass Index (BMI) of 45.7 kg/m2. Past medical history was significant for attention deficit hyperactivity disorder (ADHD), osteoarthritis (OA), chronic bilateral knee pain, chronic back pain, major depressive disorder, adult onset diabetes mellitus (AODM), gout, hyperlipidemia, hypertension, insomnia, self-reported cardiac arrythmia with patient endorsing a diagnosed bundle branch block, which do not show up in records and patient lacked knowledge about lateralization; nephrolithiasis, restless leg syndrome, scoliosis, and obstructive sleep apnea (OSA) managed with a continuous positive air-pressure machine (CPAP). Additionally, the patient reported he had undergone a cardiac stress testing two years prior, with normal results. 

The patient’s list of medications was significant for use of atorvastatin 40mg by mouth (PO) once daily (QDAY), cyclobenzaprine 5mg PO three times a day (TID), lisdexamfetamine 50mg PO QDAY, lisinopril 20mg PO QDAY, magnesium oxide 400mg PO twice a day (BID), metformin 500mg PO BID, ropinirole PO QDAY, sertraline 50mg PO QDAY, tramadol 50mg PO BID, trazodone 100mg PO QDAY, meloxicam 10mg PO QDAY, tadalafil 10mg PO when needed (PRN). The patient endorsed allergies to penicillin, azithromycin, and cephalexin, to which he reported breaking out in hives when exposed. Past surgical history was significant for scrotum exploration, a hernia repair with a groin exploration, five separate surgeries for nephrolithiasis removal, a rhinoplasty, a tonsillectomy, as well as an adenoidectomy. The patient reported no significant issues with previous surgical procedures, although notes mention laryngospasm following extubation, which was noted and kept in consideration by the anesthesiology team. The patient denies tobacco use or alcohol. The planned procedures were for lumbar radiculopathy, included a L2-L3, L3-L4 laminectomy and decompression, a right L2-L3 transforaminal lumbar interbody fusion (TLIF), a L2-L3 posterior spinal instrumented fusion (PSIF) and L2-L3 posterolateral (intertransverse) fusion. The surgical procedure had no significant issues, other than a blood loss that was estimated to be around 400-450mL. The surgeon had also utilized a large cancellous autograft from frozen viable cells. 

**Figure 1:**
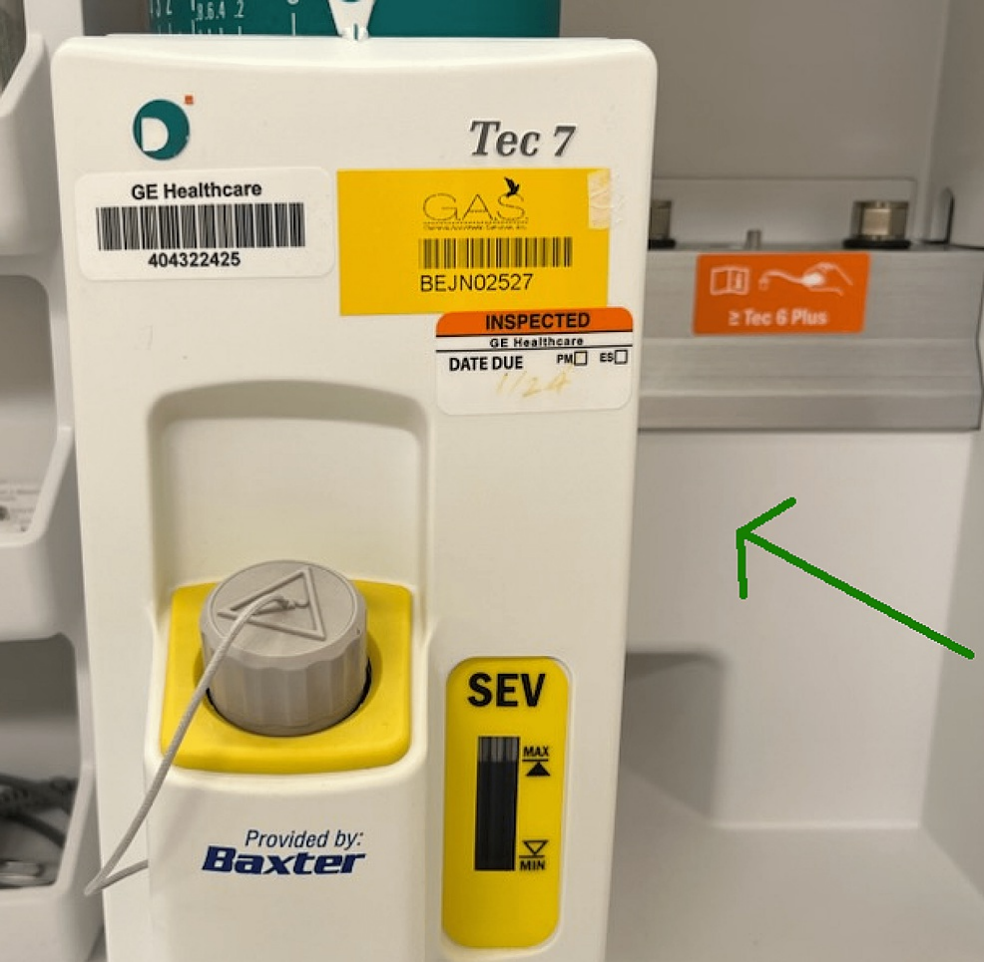
Anesthetic Vaporizer

The anesthesia plan was discussed prior to surgery with the patient, and informed consent was obtained. Once in the operating room, the patient’s airway was measured as a Mallampati III. Anesthesia included use of an isoflurane vaporizer through an endotracheal tube (Figure 1). Postoperatively, the anesthesiologist planned to do a trial extubation before being transferred to the post-anesthesia care unit (PACU). 

The surgical procedure was conducted as expected with no complications reported by the operating surgeon. However, towards the end of the procedure the patient’s overall condition started to deteriorate. The patient was reported to be hypercapnic, although exact numbers were not included in the post-operative report, and had a significant increase in his respiratory rate. It was therefore decided to increase the fraction of inspired O2 (FIO2) delivered to the respirator to 40%. The Certified Registered Nurse Anesthetist (CRNA) physically present during the case suspected malignant hyperthermia, although at no time did the patient ever have an increase in core body temperature nor did he exhibit muscle rigidity. As such, he was not given dantrolene. As a result, the patient was placed on a bilateral positive airway pressure machine (BiPAP) and was admitted directly to the ICU for postoperative management. 

Upon admission to the intensive care unit (ICU), the patient’s vitals were notable for a blood pressure of 173/83mmHg, which was reverified and found to be correct. His temperature was 36.7 degree Celsius, which was within normal limits. Initial laboratory investigations revealed the values included below (Table 1). Patient was kept on BiPAP with 40% FIO2 initially, which was progressively weaned over the course of three days. The patient was equally treated with intravenous (IV) insulin for hyperglycemia and hyperkalemia, IV normal saline and clindamycin. Repeat labs were completed, and three days later the patient was discharged. Upon time of writing, the patient did well and followed up with his primary care physician. 

**Table 1:** Initial Laboratory Investigations pCO2: partial pressure of carbon dioxide, pO2: partial pressure of oxygen

Laboratory Results	Measured Value	Expected/Standard Values
White Blood Cell Count	11,000 cells/uL	4500-11,000 cells/uL
Sodium	133 mEq/L	136-146 mEq/L
Potassium	6.3 mEq/L	3.5-5.0 mEq/L
Glucose	316 mg/dL	<140 mg/dl Random, Non-Fasting
Calcium	8 mg/dL	8.4-10.2 mg/dL
pH	7.295	7.35-7.45
pCO2	45.3 mmHg	33-45 mmHg
pO2	102.9 mmHg	75-105 mmHg

## Discussion

This patient experienced very serious and potentially life-threatening intra-operative complications, significantly outside the boundaries of what was to be expected during something considered to be a routine elective procedure. It is a stark reminder that everything we do in the medical field comes with some level of danger and the possibility, however rare, that there could be life-threatening consequences. Although a full workup was not completed to determine the exact underlying cause of this patient's rapid decline, considering he recovered very quickly once outside the OR, it is difficult to imagine based on the numerous references included above that it would not be strongly correlated with his numerous underlying health conditions and extensive pharmaceutical therapies. 

Elective procedures are medical interventions that undoubtedly are vastly beneficial overall, but also come with significant risks. Previous research indicates that the death rate amongst elective procedures in the United States is about 0.17% [9]. Although a less statistically inclined reader may wish to consider that figure minimal, patients may not truly understand the risk to them in particular, not simply some nameless patient. It is therefore vital as clinicians that we adequately explain these potential complications, ensuring that they fully understand the risk they are undertaking [10,11]. It is good to note that enormous effort has been made in recent years to research informed consent and how to better explain procedures to patients [10,11]. 

Another factor to consider is selectivity by clinicians. There have always been multiple levels of physicians between a patient and a surgery, attempting to discern who is, or isn’t, a good surgical candidate [3,4,6,12]. Nonetheless, based on the context of an aging population and worsening underlying health conditions, perhaps surgeons should be increasingly selective about which patients should receive surgical interventions, and who should be given a plan to improve overall health before intervention would be considered [3,4,6,12]. This case is a prime example of a procedure being performed on a patient with an extensive medical history and poor physical condition, two factors that undoubtedly played a role in the intraoperative crisis that occurred. The fact that he was severely obese, suffered from long-standing cardiovascular disease, and had significant polypharmacy should have resulted in a more careful approach to the management of this patient’s condition [3,4,6,12]. While orthopaedic surgery is generally safe and effective with great benefits to patients, it should not be the first-line therapy especially in more complex patient populations. 

As mentioned above, our patient was on a significant number of medications at the time of the procedure. Numerous medication interactions, physiological changes, and overall association with numerous underlying health conditions make polypharmacy a significant issue to consider before surgical intervention [13]. Of the most well-known surgical risk calculation tools, such as the Goldman index, actual number of pharmaceuticals is not included in the calculation, only the underlying health conditions these medications treat. Polypharmacy has been shown in research to drastically increase the rate of complications to surgical procedures, and therefore it should be considered in preoperative clearance whether such a procedure is warranted given the increased risk [13]. 

Another factor that should have been considered before deciding to proceed with this operation was the high body mass index of this patient. At a BMI of 45.7 kg/m2, he was morbidly obese, and it is reasonable to assume that significant portions of his pain are directly attributable to excess weight causing undue stress on his frame and joints. There is substantial research to suggest that patients that are obese or overweight have worse outcomes in surgery compared to those who are not [3,4]. While orthopaedic surgery is very useful and has improved the lives of countless individuals through surgical interventions, it should be considered that more conservative measures be prioritized or attempted first before making the decision to operate, especially considering the increased risk these patients face. As our patient population gradually gets heavier, surgeons should be careful of these confounding factors and should also strive to make the options and risk of a procedure aware to a patient to ensure informed consent. While this case ended on a positive note, it could very easily have ended in patient death, and as such should serve as a cautionary note to orthopaedic surgeons. For future publications, researchers could expand on whether plans for weight loss could have significant reduction in peri-operative risk for patients [14]. 

## Conclusions

We hope this case of a severe intraoperative complication in a patient undergoing orthopaedic laminectomy serves as a learning opportunity for all orthopaedic surgeons in pre-, intra-, and post-operative management in complicated patients. We also hope this paper will contribute to the growing body of data analyzing the effects of polypharmacy and underlying health conditions on calculation of pre-surgical risk. This patient was severely obese and on a significant amount of different medical therapies. Surgeons should also be mindful of any more conservative therapies that may be indicated to treat the patient before making the decision to operate. Additionally, we hope this paper will help further the discussion on including weight and pharmaceutical use in pre-operative risk management tools, such as the Goldman’s index, as well as consider weight loss a number of weeks before surgery, comparable to how many surgeons require patients to cease smoking eight weeks prior to major surgery. 
